# Validation of a lifestyle-based risk score for type 2 diabetes mellitus in Australian adults

**DOI:** 10.1016/j.pmedr.2021.101647

**Published:** 2021-11-18

**Authors:** Vera Helen Buss, Marlien Varnfield, Mark Harris, Margo Barr

**Affiliations:** a Australian e-Health Research Centre, Commonwealth Scientific and Industrial Research Organisation, Surgical Treatment and Rehabilitation Service – STARS, Level 7, 296 Herston Road, Herston, Queensland 4029, Australia; b Centre for Primary Health and Equity, University of New South Wales, Level 3, AGSM Building, UNSW Sydney, New South Wales 2052, Australia

**Keywords:** Diabetes mellitus, type 2, Risk factor scores, Logistic regression, Validation study, Cohort analysis

## Abstract

The study aimed to assess the performance of a lifestyle-based prognostic risk model (Diabetes Lifestyle Score) for the prediction of 5-year risk of type 2 diabetes mellitus. The model comprises nine self-reported predictors (sex, age, antihypertensive drugs, body mass index, family history of diabetes, physical activity, fruits, vegetables, and wholemeal/brown bread). We conducted an external validation and update of the model in an Australian cohort including 97,615 residents of New South Wales aged 45 years and older who were free of type 1 and 2 diabetes mellitus at baseline. Of all participants, 4,741 developed type 2 diabetes mellitus over 5 years. We conducted the statistical analyses in RStudio using the programming language R. The area under the receiver operating characteristic curve (AUC) of the original model was 0.726 (95% confidence interval: 0.719, 0.733). After adjusting the calibration intercept and slope, the original model performed reasonably well in the external cohort. The best performance was measured by using the numerical predictors as continuous variables and refitting all coefficients (AUC: 0.741, 95% confidence interval: 0.734, 0.748). The results of the original model after calibration were comparable to those received from the AUSDRISK score which is routinely used in Australian clinical practice. Hence, the lifestyle-based model might be a reasonable alternative for laypersons since the required information is most likely known by these. Further, the risk score may communicate the message about the importance of a healthy diet to reduce the risk of diabetes.

## Highlights

• A lifestyle-based score can satisfactorily predict 5-year risk of type 2 diabetes.
• The model’s performance was similar to the standard tool in an Australian cohort.
• Lifestyle predictors might be easier for laypersons to know and interpret.

## 1 Introduction

### 1.1 Medical context

The progression to diagnosed type 2 diabetes mellitus (T2DM) is associated with unhealthy lifestyle factors, such as lack of physical activity, sedentary behaviour, and poor diet (GBD 2017 Risk Factor Collaborators, 2018). Based on self-reported data from the National Health Survey (Australian Bureau of Statistics, 2019), almost 1 million Australians, which represents 4.1% of the population, had T2DM in 2017–18. The same survey showed that for those aged 18 years and older 66.4% were either overweight or obese, 94.8% had inadequate fruit or vegetable intake, and 84.6% did not meet guidelines for physical activity (Australian Bureau of Statistics, 2019). In a systematic review, Glechner et al. (Glechner et al., 2018) demonstrated in a pooled analysis of 16 randomised controlled trials the effectiveness of lifestyle-based interventions in lowering the progression rate from pre-diabetes to T2DM. In an attempt to stop the increasing prevalence of T2DM it is vital to identify individuals at risk and, subsequently, offer them appropriate preventative treatment.

### 1.2 Rationale for external validation

In 2016 Abbasi et al. (Abbasi et al., 2012) conducted a systematic review of risk models for T2DM. They found 16 development studies for T2DM incidence. In 2011, Noble et al. (Noble et al., 2011) identified 145 prognostic risk models and scores. Despite the abundance of models, the authors argued that many have been developed without any practical application in mind. Risk scores commonly used in clinical practice, such as the Framingham diabetes risk calculator (Wilson et al., 2007) or the AUSDRISK score (Chen et al., 2010), face the problem that laypersons might not be able to determine their risk using these scores because they require information that laypersons might not know such as lipid levels or history of high blood glucose. Simmons et al. (Simmons et al., 2007) developed a simple lifestyle-based risk score (from here onwards called ‘Diabetes Lifestyle Score’) using data from the European Prospective Investigation into Cancer and Nutrition (EPIC)-Norfolk study (Day et al., 1999). To our knowledge, there is no published external validation of the model in the Australian setting. Hence, its performance in the Australian population is unknown.

### 1.3 Performance metrics

The Brier score is a quadratic scoring rule for binary outcomes and is a measure of overall performance (calibration and sharpness) (Brier, 1950, Rufibach, 2010). The calibration of the model is preferably assessed with a graph; in large sample sizes, quantitative measures such as the Hosmer-Lemeshow test are almost always statistically significant (Kramer and Zimmerman, 2007, Moons et al., 2015). The calibration curve shows the predicted proportion according to the model against the observed proportion with the outcome of interest. It explains how well a model’s outcome predictions match the observed outcomes (Moons et al., 2015). Deviations of the fitted line from the ideal line indicate miscalibration, either by under- or over-estimating risk (fitted curve above or below the ideal line, respectively). Discrimination describes a model’s ability to differentiate between individuals who experience the outcome from those who do not (Moons et al., 2015). It can be assessed by plotting the false positives (1-specificity) against the true positives (sensitivity). This graph is called the receiver operating characteristic curve (ROC). The area under the curve (AUC) is a qualitative measure of discrimination. The AUC can range from 0.5 to 1, with 0.5 indicating that the model’s ability to predict the outcome is random, while 1 indicates perfect outcome prediction (Harrell, 2015).

### 1.4 Objective

This study aimed to externally validate and update the Diabetes Lifestyle Score for the prediction of T2DM in a cohort of Australians aged 45 years and older.

## 2 Methods

We followed the transparent reporting of a multivariable prediction model for individual prognosis or diagnosis (TRIPOD) statement by Collins et al. (Collins et al., 2015). Ethics approval for the 45 and Up Study was provided by the University of New South Wales Human Research Ethics Committee (HREC). This study has been approved by the New South Wales (NSW) Population & Health Services Research Ethics Committee (HREC/16/CIPHS/14) and the CSIRO Health and Medical Human Research Ethics Committee (2021_018_RR).

### 2.1 Derivation dataset and risk model

The EPIC Norfolk study is a prospective cohort study including patients aged 40 to 79 years of age from general practices in the Norfolk region of the United Kingdom (Simmons et al., 2007). Recruitment took place between 1993 and 1998. Of the 77,630 people invited, 25,633 consented and attended the baseline health check; this corresponded to a response rate of 33% (Simmons et al., 2007). In the baseline survey, data were collected on health and lifestyle as well as diet-specific data via a semi-structured food frequency questionnaire. Between 1998 and 2000, 15,028 participants undertook a follow-up health check, which corresponded to a retention rate of 58.6% (Simmons et al., 2007). At baseline, 583 individuals were identified as having diabetes. These were excluded from the analysis. The remaining participants (n = 25,038) were randomly split into training and test datasets while ensuring an equal distribution of diabetes incidence during follow-up through stratification (Simmons et al., 2007). During a mean follow-up time of 4.6 years (range 2–7 years), 417 individuals (1.7%) developed T2DM. Diabetes diagnosis was assessed using data from the follow-up health checks, hospital and general practice registers, prescription of antidiabetic medication, and baseline or follow-up data on glycated haemoglobin levels (Simmons et al., 2007).

**Fig. 1:**
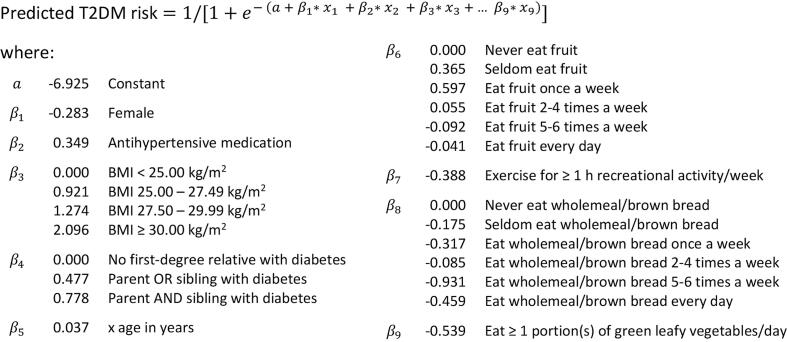
Diabetes Lifestyle Score according to Simmons et al. (Simmons et al., 2007). Abbreviations: BMI = body mass index, T2DM = type 2 diabetes mellitus.

The Diabetes Lifestyle Score (Fig. 1) is a multivariable logistic regression model developed by Simmons and colleagues (Simmons et al., 2007). The predictors are sex, age, family history of diabetes, use of antihypertensive drugs, body mass index (BMI), physical activity, and diet (green leafy vegetables, fruits, wholemeal/brown bread). The outcome is the incidence of T2DM during follow-up.

### 2.2 Validation cohort

The Sax Institute’s 45 and Up Study is a prospective cohort study including residents of NSW, Australia, who were aged 45 years and older at recruitment (Sax Institute, 2019a). The study collaborators published a detailed study description (45 and Up Study collaborators, 2008). The recruitment phase was from 2006 to 2009. The first wave of follow-up took place between 2012 and 2015 (Sax Institute, 2019a). The study comprises a total of 267,153 participants (Sax Institute, 2019a). The recruitment process was facilitated through the Services Australia (formerly the Australian Government Department of Human Services and Medicare Australia) Medicare enrolment database by contacting a random sample of the population (stratified by two age groups and two regions). People over the age of 80 years and residents of rural and remote areas were oversampled. The response rate was 18% which represented about 11% of the NSW population aged 45 years and older. The baseline and follow-up questionnaires included information on lifestyle behaviour, medical history, family history of chronic diseases, socioeconomic status, and geographic factors (Sax Institute, 2019a). The 45 and Up Study questionnaire data were linked deterministically to the Pharmaceutical Benefits Scheme (PBS; prescribed drugs) data. The linkage was facilitated by the Sax Institute using a unique identifier provided by Services Australia. The Centre for Health Record Linkage (CHeReL, 2021) linked the records probabilistically to the NSW Admitted Patient Data Collection (APDC; hospital data), the NSW Register of Births, Deaths & Marriages – Death Registrations (mortality), and the Australian Bureau of Statistics (ABS) mortality data (cause of death unit record files).

### 2.3 Assessment of outcome

We used a similar method to the one described by Comino et al. (Comino et al., 2013) to assess the incidence of T2DM. First, we excluded all participants with a diagnosis of type 1 or T2DM at baseline from further analysis. Women remained in the dataset if they were classified as having had gestational diabetes, but no further history of diabetes was reported. Gestational diabetes was classified based on the age of the diabetes diagnosis and the age of the last delivery, both self-reported in the baseline questionnaire. A woman was classified as having had gestational diabetes if she received the diabetes diagnosis before the date of her last delivery and if there was no report of diabetes medication on the baseline questionnaire and in the PBS data of the previous 12 months. We assumed that everyone who developed diabetes after baseline would have developed T2DM which is consistent with the study by Thunander et al. (Thunander et al., 2008) showing that 94% of new diabetes mellitus cases in people aged 40–100 years is T2DM. We identified T2DM cases from the 45 and Up Study baseline and follow-up questionnaire via question 23 (medications in last four weeks: Diabex, Diaformin, or Metformin) and question 24 (*“Has a doctor EVER told you that you have diabetes?”*). We identified diabetes-related hospital admissions before baseline using the ICD-10-AM (international statistical classification of disease and related health problems, 10th revision, Australian modification) codes E10-E14 and O24.0-O24.9 (Australian Institute of Health and Welfare, 2020). These comprise all types of diabetes mellitus. For the time between baseline and follow-up, we included only the ICD-10-AM codes E11 and O24.1 which correspond to T2DM only. We searched the PBS data for all claims related to diabetes medication (such as insulin and other blood-glucose-lowering drugs) and diagnostic agents (such as sensors and strips). To adjust for changes over time, we included PBS item codes of listings from three different years (2003, 2009, and 2020) (Australian Government Department of Health, 2020a, Australian Government Department of Health, 2020b, Australian Institute of Health and Welfare, 2009, Commonwealth of Australia, 2003).

### 2.4 Assessment of predictors

The predictor variables are all from the 45 and Up Study baseline survey. We calculated BMI after imputing missing values for height and weight. Before the imputation, we removed height and weight values if they resulted in BMI values below 9 and above 50 as these are considered invalid in the 45 and Up Baseline Data Dictionary (Sax Sax Institute, 2013).

### 2.5 Missing values

We looked for any patterns of missingness to draw inferences about the type of missing data. Then, we imputed missing values using the MICE (multivariate imputation by chained equations) package in R (van Buuren and Groothuis-Oudshoorn, 2011). The multiple imputation process included all predictor variables (sex, age, antihypertensive medication, height, weight, father/mother/siblings with diabetes, moderate/vigorous physical activity, serves of cooked/raw vegetables, serves of fruits, slices of brown bread) as well as the outcome variable (T2DM at follow-up). Binary variables (sex, antihypertensive medication, father/mother/siblings with diabetes) were handled as factors, all others as numeric variables. For the imputation, we used the function’s default settings (i.e., five imputations; predictive mean matching for numeric data; logistic regression imputation for binary data; five iterations). We estimated regression coefficients using all five imputations before pooling the results. To assess model performance, we used the data of the first imputation.

### 2.6 Statistical analyses

We tested for statistically significant differences between the derivation and validation cohorts by computing Pearson’s χ^2^ test with Yates’ continuity correction to compare proportions and the Welch’s *t*-test to compare the age distributions. We assessed the original model as published by Simmons et al. (Simmons et al., 2007), two recalibrated models, and three refitted models (see Table 1), according to the methods described by Janssen et al. (Janssen et al., 2008). We tested the significance of the predictors in the refitted model by computing the likelihood ratio test. We set the significance level for all statistical tests to 0.05.

**Table 1:** Updating methods for the logistic regression model.

**Method**	**Description**
0 – no adjustments	see Fig. 1
1 – calibration-in-the large	adjust intercept based on T2DM incidence in the validation dataset
2 – logistic calibration	adjust intercept and regression coefficients using calibration intercept and slope from logistic regression model fitted with linear predictor as the only covariate
3 – refitting	re-estimate all regression coefficients using only the validation dataset
4 – refitting with different predictor assessment	like 3, but with overall vegetable consumption (cooked + raw vegetables) as a proxy for green leafy vegetables instead of raw vegetables
5 – refitting with numerical predictors as continuous	like 4, but numerical predictors (BMI, moderate + vigorous physical activity, raw + cooked vegetables, fruits, brown bread) as continuous variables

Abbreviations: BMI = body mass index, T2DM = type 2 diabetes mellitus.

To assess the models’ performance, we determined discrimination, calibration, and overall model performance using the Brier score. For discrimination, we calculated AUC and the corresponding 95% confidence interval (CI) with the roc-function from Robin’s pROC package in R (Robin et al., 2011). To assess the optimism-corrected predictive accuracy of the refitted models, we performed bootstrapping with 1000 repetitions as described by Harrell et al. (Harrell et al., 1996). We compared the results among the models and to the AUC of the original Diabetes Lifestyle Score in the derivation data reported by Simmons et al. (Simmons et al., 2007). For the calibration curve, we used the val.prob-function from Harrell’s rms package (Harrell, 2020) which includes a smoothed line computed with the loess algorithm (Austin and Steyerberg, 2014). We computed the Brier score also with the val.prob-function. For better interpretability, we scaled the score by its maximum (Brier_scaled_ = (1 – Brier/Brier_max_)*100, where Brier_max_ is 0.0475 at an incidence rate of 5%) to have percentage values ranging from 0 to 100% (ideal) (Steyerberg, 2019).

**Fig. 2:**
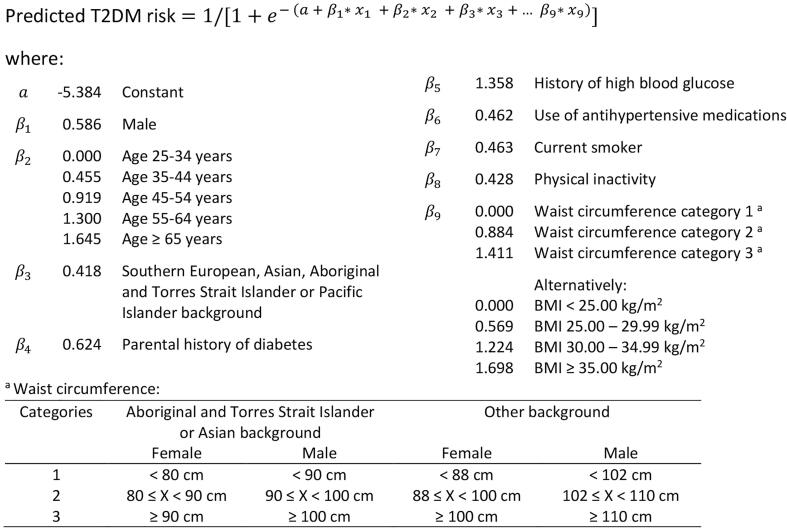
Logistic regression model of AUSDRISK score (Chen et al., 2010). **Abbreviations: BMI = body mass index, T2DM = type 2 diabetes mellitus.**

We compared the results to the AUSDRISK tool (Fig. 2) which is the model that is used in Australian clinical practice to predict the risk of T2DM in next the five years (Chen et al., 2010). We externally validated a modified version of the model in the validation dataset following the methods outlined above.

### 2.7 Software

We conducted the analysis in RStudio (Version 1.2.5042) (RStudio Team, 2020) using the programming language R (Version 4.0.0) (R Core Team, 2020). The validation datasets are stored in the Secure Unified Research Environment (Sax Institute, 2019b).

## 3 Results

### 3.1 Participants

**Fig. 3:**
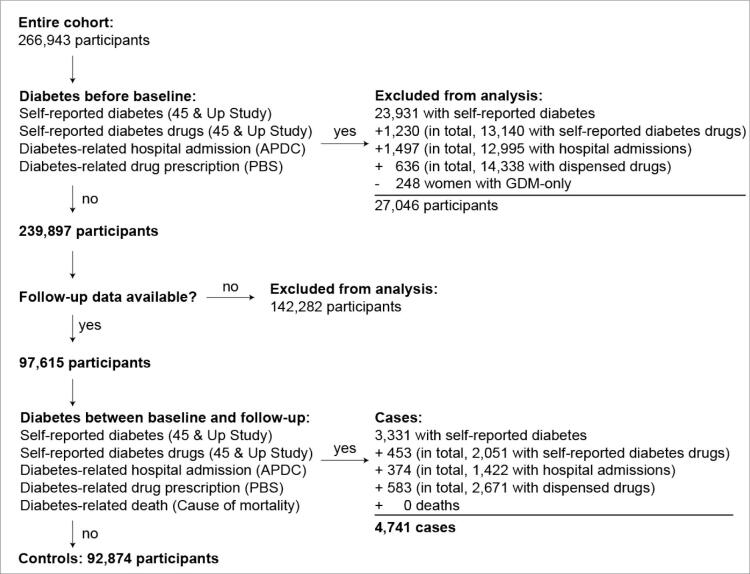
Flowchart for identifying T2DM cases and controls. APDC = Admitted Patient Data Collection data; GDM = gestational diabetes mellitus; PBS = Pharmaceutical Benefits Scheme data.

At baseline, we had access to data of 266,943 participants. Of these, 27,046 participants were excluded because they were classified as having type 1 or T2DM. Follow-up information was available for 97,615 participants who did not have diabetes mellitus at baseline. Of these, 4,741 participants were classified as having T2DM at the scheduled 5-year follow-up. This represents an incidence rate of 4.9%. Fig. 3 shows a flowchart detailing the process of participant selection and outcome assessment. At baseline, the median age of participants who were included in the analysis was 59.1 [interquartile range (IQR): 13.9] years. Fifty-seven percent were female. The mean scheduled 5-year follow-up time for all participants was 5.7 [standard deviation (SD): 1.5] years. For cases, i.e., participants with T2DM at follow-up, the mean time was 6.0 (SD: 1.7) years, and for controls, i.e., participants without T2DM at follow-up, 5.7 (SD: 1.5) years. The total follow-up time for all participants was 556,783 years. There were significant differences between the baseline demographics of the derivation and validation cohorts (Table2); the direction of the trends between people with diabetes and without diabetes was the same.

**Table 2:** Comparison of participants’ characteristics in derivation (Simmons et al., 2007) and validation cohort.

**Variables**	**With diabetes**		**Without diabetes**		**p-value**^d^
	*Derivation*	*Validation*	*Derivation*	*Validation*	
All respondents a	209 (1.7)	4,741 (4.9)	12,310 (98.3)	92,874 (95.1)	<0.001
Age (in years) b	62.8 (8.4)	62.4 (9.3)	59.0 (9.3)	60.2 (9.6)	<0.001
Women a	92 (44.0)	2,279 (48.1)	6,842 (55.6)	53,005 (57.1)	<0.001
Family history a					<0.001
Parent or sibling with diabetes	32 (15.3)	1,352 (28.5)	1,362 (11.1)	16,978 (18.3)	
Parent and sibling with diabetes	5 (2.4)	245 (5.2)	106 (0.9)	1,940 (2.1)	
Body mass index a					<0.001
< 25.0	25 (12.1)	725 (16.4)	4,980 (40.5)	35,941 (41.3)	
25.0–27.5	51 (24.6)	805 (18.2)	3,392 (27.6)	20,684 (23.7)	
27.5–30.0	48 (23.2)	872 (19.7)	2,141 (17.4)	14,393 (16.5)	
> 30.0	83 (40.1)	2,031 (45.8)	1,772 (14.4)	16,074 (18.5)	
Antihypertensive drugs a	66 (31.6)	1,708 (36.0)	2,196 (17.8)	18,253 (19.7)	<0.001
Physical activity ≥ 1 h/week a	57 (27.3)	3,291 (73.0)	5,782 (47.0)	72,076 (80.9)	<0.001
Green leafy (raw) c vegetables ≥ 1 portion/day	28 (13.5)	3,480 (85.1)	2,485 (20.6)	72,470 (87.5)	<0.001
Fresh fruits ≥ 1 portion/day a	83 (40.5)	4,119 (91.9)	6,006 (49.7)	83,341 (93.4)	<0.001
Wholemeal/brown bread ≥ 1 portion/day a	64 (32.2)	3,832 (86.0)	4,698 (39.8)	78,033 (87.7)	<0.001

a n (%).

b mean (standard deviation).

c in derivation dataset: green leafy vegetables; in validation dataset: raw vegetables.

d differences between derivation and validation cohort, for age Mann-Whitney *U* test and all other variables Pearson’s χ^2^ test with Yates’ continuity correction.

### 3.2 Missing values

Complete data were available for 76.0% of participants. The most frequently missing variable was serves of raw vegetables, in 11.0% of participants. Table3 summarises the proportion of missing values for each variable. The highest number of missing values per participant was six, which applied to 11 participants. The most common combination of missing predictors was concerning food serves (fruits, slices of brown bread, cooked and raw vegetables), which occurred in 1,065 participants (1.1%). Participants with complete data were, on average, less likely to develop diabetes (4.6% vs. 5.7%, p < 0.001), younger (median age 59 years vs. 61 years, p < 0.001), more likely to be female (58.0% vs. 52.5%, p < 0.001), less likely to be overweight or obese (p < 0.001), less likely to take antihypertensive drugs (20.3% vs. 21.0%, p = 0.023), more likely to exercise for at least one hour per week (82.0% vs. 74.9%, p < 0.001), more likely to eat at least one serve of cooked vegetables per day (97.8% vs. 98.3%, p < 0.001), more likely to eat at least one serve of fruits per day (93.6% vs. 92.6%, p < 0.001), more likely to eat at least one slice of brown bread every day (88.3% vs. 85.0%, p < 0.001), and had a slightly different likelihood of a family history of diabetes (p = 0.038). Before imputing missing values using MICE, we set missing values for fruit and vegetable serves to zero if the participants stated in the questionnaire that they did not eat any fruit or vegetables, respectively. This reduced the percent of missing values for fruits to 3.0%, for raw vegetables to 10.7%, and for cooked vegetables to 2.9%.

**Table 3:** Percent of missing values per predictor.

**Predictor**	**Percent (%) of missing values**
Sex	0.0
Age	0.0
Family history	0.0
BMI a	6.2
Antihypertensive drugs	0.0
Physical activity	4.2
Raw vegetables	11.0
Cooked vegetables	3.1
Fruits	4.0
Brown bread	4.3

a weight 3.3% and height 4.8% missing values.

### 3.3 Performance of the original model

Using the original model (only changing green leafy vegetables to raw vegetables), the AUC was 0.726 (95% CI: 0.719, 0.733) and the scaled Brier score was 1.47% (Table 4). The AUC reported in the original study using the derivation dataset was 0.762 (95% CI: 0.730, 0.790) (Simmons et al., 2007). After recalibrating the model by adjusting the intercept only, the scaled Brier score changed to 5.26%. Logistic calibration resulted in a scaled Brier score of 5.89%.

**Table 4:** Overview of models’ discrimination and overall performance in the validation.

**Method/model**	**AUC (95% CI)**	**AUC_bias_ (95% CI)**	**Brier_scaled_**	**Slope (95% CI)**	**Intercept (95% CI)**
0 – no adjustments	0.726 (0.719, 0.733)	–	1.47%	0.781 (0.752, 0.811)	0.669 (0.539, 0.800)
1 – calibration-in-the-large	0.726 (0.719, 0.733)	–	5.26%	0.781 (0.752, 0.811)	−0.531 (−0.618, −0.444)
2 – logistic calibration	0.726 (0.719, 0.733)	–	5.89%	1.000 (0.962, 1.038)	0.000 (−0.106, 0.106)
3 – refitting	0.738 (0.731, 0.745)	0.737 (0.731, 0.744)	6.53%	1.000 (0.965, 1.035)	0.000 (−0.098, 0.098)
4 – refitting with different predictor assessment	0.738 (0.731, 0.745)	0.737 (0.731, 0.745)	6.53%	1.000 (0.965, 1.035)	0.000 (−0.098, 0.098)
5 – refitting with numerical predictors as continuous	0.741 (0.734, 0.748)	0.741 (0.734, 0.748)	6.53%	1.000 (0.966, 1.034)	0.000 (−0.097, 0.097)
AUSDRISK	0.723 (0.716, 0.730)	–	4.42%	0.956 (0.920, 0.991)	−0.514 (−0.600, −0.430)

Abbreviations: AUC = area under the receiver-operator curve; AUC_bias_ = bias-corrected AUC for refitted models; Brier_scaled_ = scaled Brier score; CI = confidence interval.

### 3.4 Specifications of updated models

Sex, age, antihypertensive drugs, BMI, family history, and physical activity were statistically significant predictors in all the refitted models (likelihood ratio test, Table5). Brown bread was not statistically in any of the refitted models. Fruit and vegetables (if raw only and if combined) were statistically significant predictors if categorised but not as a continuous variable.

**Table 5:** Results of likelihood ratio test for refitted models (in sequential order).

**Variables**	**Refitted, categorised**			**Refitted, continuous**		
	*deviance*	*df*	*p-value*	*deviance*	*df*	*p-value*
Sex	147.38	1	<0.001	147.38	1	<0.001
Age	190.60	1	<0.001	190.60	1	<0.001
Antihypertensive drugs	516.25	1	<0.001	516.25	1	<0.001
BMI	1986.03	3	<0.001	2033.14	1	<0.001
Family history	404.05	2	<0.001	408.56	2	<0.001
Physical activity	49.68	1	<0.001	31.10	1	<0.001
Fruits	7.91	2	0.019	3.49	1	0.062
Vegetables a	6.05	1	0.014	2.54	1	0.111
Brown bread	3.15	4	0.533	0.49	1	0.484

Abbreviation: df = degrees of freedom.

a raw and cooked vegetables combined.

### 3.5 Performance of the updated models

**Fig. 4:**
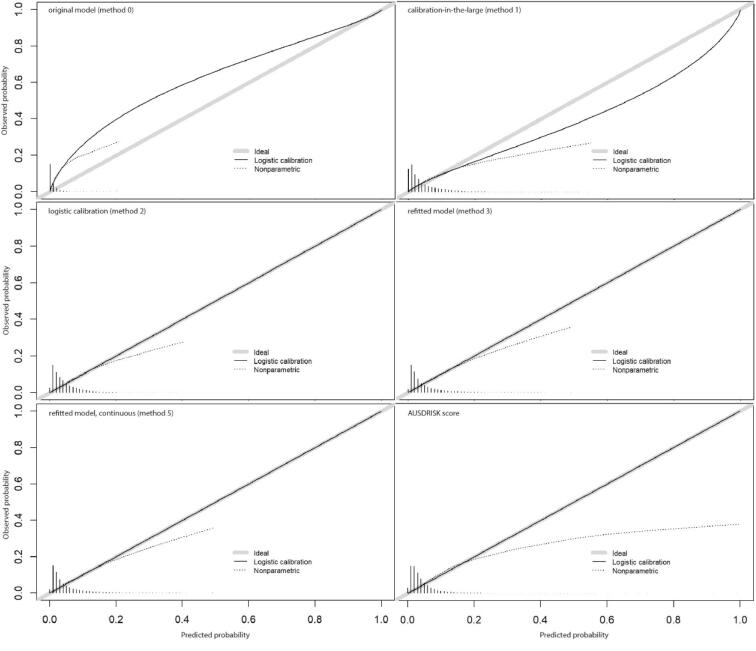
Calibration curves, vertical lines indicate the predicted probability distribution.

The AUC varies from 0.726 (95% CI: 0.719, 0.733) for the original model to 0.742 (95% CI: 0.735, 0.749) for the refitted model with continuous variables (Table4). The scaled Brier scores are all relatively low which indicated that the overall performance of the models is low. The calibration curve of the original model shows that the predicted risk underestimated the observed risk (Fig. 4). After recalibration, in the non-parametric model, the predicted risk appears to slightly overpredict the risk, especially for the high-risk groups. The AUSDRISK model showed acceptable discrimination (Table 4) and calibration (Fig. 4) without adjustments. The AUC and scaled Brier score of the AUSDRISK score are similar to those of the Diabetes Lifestyle Score without adjustments.

## 4 Discussion

### 4.1 Interpretation

This study externally validated and updated the Diabetes Lifestyle Score for the prediction of T2DM incidence within five years in a linked dataset including the 45 and Up Study cohort. Even though the baseline demographics of the derivation and the external validation cohorts differed, the original model shows good discrimination in the external dataset [AUC of 0.726 (95% CI: 0.719, 0.733)]. The model performance can be slightly improved by recalibration. Further refitting of the model did not lead to meaningful improvements. The consumption of brown bread and vegetables did not have considerable weight in the prediction models. By comparing the discrimination and calibration of the Diabetes Lifestyle Score with the AUSDRISK tool in the 45 and Up Study, the former had better discrimination [AUC: 0.726 (95% CI: 0.719, 0.733) vs. AUC: 0.723 (95% CI: 0.716, 0.730)] and a comparable calibration after adjusting slope and intercept. In Australia, the AUSDRISK tool by Chen et al. (Chen et al., 2010) is the model used in clinical practice. Chen et al. (Chen et al., 2010) performed two external validations, using the Blue Mountains Eye Study (BMES) and the North West Adelaide Health Study (NWAHS). The AUSDRISK tool was slightly modified to adjust for the variables available in the external datasets. The resulting AUCs were 0.66 (95% CI: 0.60, 0.71) using BMES compared to 0.75 (95% CI: 0.72, 0.78) by applying the same modified model to the Australian Diabetes Obesity and Lifestyle (AusDiab) study in which the model was developed, and 0.79 (95% CI: 0.72–0.86) using NWAHS compared to 0.79 (95% CI: 0.76, 0.82) in the AusDiab study. In our external validation, we used the same modified version that was used for the BMES. In comparison, the AUSDRISK score achieved better discrimination in the 45 and Up Study, and calibration was good, too.

### 4.2 Strengths and limitations

An important strength of this study is that we followed the TRIPOD statement. We performed the analysis in a large cohort study, and we used bootstrapping to correct for optimism in the refitted models. Among the limitations are that the dataset contained missing values, particularly in diet-related variables, and that the predictor assessment and part of the outcome assessment were based on self-reported data. However, if laypersons used the risk score, it is to be expected that some of the bias introduced through self-reporting would also be inherent in the information these provided when calculating their risk. Ng et al. (Ng et al., 2011) who investigated the bias introduced through self-reported height and weight in the 45 and Up Study concluded that the provided values resulted in valid measures to calculate BMI but underestimated overweight and obesity. We tried to minimise the bias introduced through missing values by using different imputation techniques. The response rate in the baseline survey was 18% and in the follow-up survey 65%. However, based on analyses conducted by Mealing et al. (Mealing et al., 2010) and Wang et al. (Wang et al., 2017), we neither believe that non-response significantly influenced the analysis nor that it affected the interpretation of our results. Further limitations of the study are that the 45 and Up Study did not collect information on some of the required predictors (for lifestyle score: green leafy vegetables, for AUSDRISK tool: history of high blood glucose and waist circumference). However, we assessed the Diabetes Lifestyle Score when using only raw or raw and cooked vegetables combined, and for the AUSRISK score, we compared our results to the results by Chen et al. (Chen et al., 2010) when using the same modified version of the score. Further, although Aboriginal and Torres Strait Islander status is collected as part of the 45 and Up Study questionnaire, we did not have access to it as part of our ethics approval. This might have resulted in a poorer model performance of the AUSRISK tool, however, the proportion of participants with Aboriginal and Torres Strait Islander or Pacific Islander status in the 45 and Up Study is low (Sax Institute, 2011).

### 4.3 Implications

The Diabetes Lifestyle Score might be an alternative to the AUSDRISK score that is currently used in Australian clinical practice, specifically for laypersons who are unable to answer some of the questions asked in the AUSDRISK score, such as history of high blood glucose. Also, when laypersons were to use the Diabetes Lifestyle Score, they might realise the importance of diet in T2DM risk; by choosing a diet rich in wholemeal, vegetables, and fruits, they can reduce their risk. For the same reason, the online version of the AUSDRISK score provided on the website of the Australian government contains a question about fruit and vegetable intake, even though these are not significant predictors and were hence removed during the model development process (Chen et al., 2010). The Diabetes Lifestyle Score could be part of a mobile health app and in this way be made available to the general population. The app could in turn form part of a health promotion program that increases awareness of diabetes risk and encourages users to take up a healthier lifestyle.

## 5 Conclusions

The lifestyle-based risk model performed reasonably well in the external validation using an Australian cohort study, especially after logistic calibration. Beyond that, refitting methods did not lead to noteworthy improvements. Additionally, in the 45 and Up Study, the performance of this lifestyle-based risk model appears to be comparable to the in Australia widely used AUSDRISK tool. That means that the lifestyle-based risk model might be a reasonable alternative for use by laypersons since the required information is most likely known by these and it may convey an important public health message about the importance of diet to those who use the risk score.

### CRediT authorship contribution statement

**Vera Helen Buss:** Conceptualization, Methodology, Software, Formal analysis, Investigation, Writing – original draft, Visualization. **Marlien Varnfield:** Conceptualization, Writing – review & editing, Supervision. **Mark Harris:** Conceptualization, Writing – review & editing, Supervision. **Margo Barr:** Conceptualization, Methodology, Validation, Resources, Data curation, Writing – review & editing, Supervision.

## Declaration of Competing Interest

The authors declare that they have no known competing financial interests or personal relationships that could have appeared to influence the work reported in this paper.

## Appendix A Supplementary data

Supplementary data 1

- Detailed description of the methods used to assess the outcome and predictors (pages 2–7).
- Background information to statistical analysis methods and software (pages 8–9), including:
  o details on the updating methods,
  o complete list of all R packages used in the analysis.
- Analysis of missing data patterns (pages 10–12).
- Details on models and their performance (pages 13–17), including:
  o receiver-operator curve of the models (Figs. A.4-A.7),
  o estimated coefficients of the updated models (Table A.7).
