# Supplementary material for: Validation of a lifestyle-based risk score for type 2 diabetes mellitus in Australian adults
Source: Prev Med Rep. 2021 Nov 18;24:101647. doi: 10.1016/j.pmedr.2021.101647 (PMC8684002; doi:10.1016/j.pmedr.2021.101647)
Supplement: Supplementary data 1 [file mmc1.docx]

Appendix

**Article**: Validation of lifestyle-based risk prediction model for type 2 diabetes mellitus in Australian adults

Table of Contents

[1. Methods 2](#_Toc72335809)

[Assessment of outcome 2](#_Toc72335810)

[Assessment of predictors 5](#_Toc72335811)

[Statistical analysis methods and software 7](#_Toc72335812)

[2. Results 9](#_Toc72335813)

[Missing values 9](#_Toc72335814)

[Performance of original model 13](#_Toc72335815)

[Specifications of updated models 13](#_Toc72335816)

[Performance of the refitted models 15](#_Toc72335817)

[3. References 17](#_Toc72335818)

# Methods

## Assessment of outcome

The definitions of type 2 diabetes mellitus and the corresponding ICD-10-AM (international statistical classification of disease and related health problems, 10^th^ revision, Australian modification) codes used are in line with the definitions used by the Australian Institute of Health and Welfare (AIHW) (Australian Institute of Health and Welfare, 2020). Table S1 summarises this information.

Table A.1: Diabetes mellitus and corresponding ICD-10-AM codes.

| **Disease** | **ICD-10-AM code** |
| --- | --- |
| Diabetes | E10-E14, O24.0-O24.9 |
| - Type 1 diabetes mellitus | E10, O24.0 |
| - Type 2 diabetes mellitus | E11, O24.1 |
| - Other and unspecified diabetes mellitus | E13, E14, O24.2, O24.3, O24.5, O24.9 |
| - Diabetes mellitus arising during pregnancy | O24.4 |

Abbreviation: ICD-10-AM = international statistical classification of disease and related health problems, 10th revision, Australian modification

Table S2 shows the diabetes drugs included in the outcome assessment including the ATC-codes (anatomical therapeutic chemical classification), active ingredients, brand names, and Pharmaceutical Benefits Scheme (PBS) item codes. The information included in the table has been collected from the PBS website (Australian Government Department of Health, 2020a), the most current PBS listing (Australian Government Department of Health, 2020b), a report by the AIHW from 2009 (Australian Institute of Health and Welfare, 2009), and a PBS listing from 2003 (Commonwealth of Australia, 2003).

Table A.2: Type 2 diabetes mellitus medication in Australia.

| **ATC-code** | **Active ingredient** | **PBS item code** |
| --- | --- | --- |
| A10A Insulin and Analogues | Insulin aspart | 11705C, 11706D, 8435Y, 8571D |
|  | Insulin glulisine | 1921D, 9224L |
|  | Insulin lispro | 11645X, 8084L, 8212F |
|  | Insulin neutral human | 1531N, 1762R |
| A10B Blood glucose lowering drugs, excl. insulins | Metformin | 12104C, 1801T, 2430X, 3439B, 8607B, 9435N |
|  | Glibenclamide | 2939Q |
|  | Gliclazide | 2449X, 8535F, 9302N |
|  | Glimepride | 8450R, 8451T, 8452W, 8533D |
|  | Glipizide | 2440K |
|  | Acarbose | 8188Y, 8189B |
|  | Pioglitazone | 8691K, 8692L, 8693M, 8694N, 8695P, 8696Q |
|  | Alogliptin | 2993J, 2944Y, 2986E |
|  | Linagliptin | 11280Q, 3387G |
|  | Saxagliptin | 10128C, 11292H, 11311H, 8983T |
|  | Sitagliptin | 11572C, 11573D, 11576G, 9180E, 9181F, 9182G |
|  | Vildagliptin | 3415R |
|  | Dulaglutide | 11364D |
|  | Exenatide | 10888C, 3423E, 3424F |
|  | Semaglutide | 12075M, 12080T |
|  | Dapagliflozin | 10011X, 11291G |
|  | Empagliflozin | 10202Y, 10206E, 11281R, 11314L |
|  | Ertugliflozin | 11570Y, 11571B, 11577H, 11585R |
|  | Rosiglitazone | 8687F, 8688G, 8689H, 8690J |
|  | Alogliptin + Metformin | 10032B, 10033C, 10035E |
|  | Dapagliflozin + Metformin | 10510E, 10515K, 10516L, 11270E, 11300R, 11313K |
|  | Empagliflozin + Linagliptin | 11269D, 11298P, 11303X, 11310G |
|  | Empagliflozin + Metformin | 10626G, 10627H, 10633P, 10639Y, 10640B, 10649L, 10650M, 10677Y |
|  | Ertugliflozin + Metformin | 11562M, 11563N, 11564P, 11568W, 11569X, 11575F, 11581M, 11584Q |
|  | Ertugliflozin + Sitagliptin | 11561L, 11578J, 11579K, 11583P |
|  | Linagliptin + Metformin | 10035H, 10044P, 10045Q, 11274J, 11282T, 11294K |
|  | Metformin + Glibenclamide | 8810Q, 8811R, 8838E |
|  | Saxagliptin + Dapagliflozin | 11286B, 11305B |
|  | Saxagliptin + Metformin | 10048W, 10051B, 10055F, 11285Y, 11299Q, 11312J |
|  | Sitagliptin + Metformin | 10089B, 10090C, 11566R, 11574E, 11580L, 11582N, 11586T, 9449H, 9450J, 9451K |
|  | Vildagliptin + Metformin | 5474D, 5475E, 5476F |
| V04 Diagnostic agents | Disc electrode sensor | 8176H |
|  | Electrode strips | 2915K, 2926B, 8522M, 8573F, 8608C, 2891E, 8557J, 8634K, 8723D, 8766J, 8749L |
|  | Reagent strips | 2890D, 2914J, 2917M, 2919P, 8053W, 8190C, 8739Y, 8759B |

Abbreviations: ATC = anatomical therapeutic chemical classification; PBS = Pharmaceutical Benefits Scheme.

**New South Wales Admitted Patient Data Collection (hospital data):**

Identified from all diagnostic fields using ICD10-AM codes (E10-E14, and O24.0-O24.4, O24.9) and were classified as Type 1, Type 2, and other type of diabetes (see Table S1).

**PBS data (medication):**

Prescribed medications based on PBS item codes provided in Table S2. Medications provided to war veterans are included in the PBS data for the period 2013-2019. According to Comino et al. (Comino et al., 2013), 6,303 participants reported possessing a Department of Veteran Affairs card.

**Australian Bureau of Statistics cause of mortality data:**

Cause of death, ICD-10-AM code E11.

## Assessment of predictors

Table A.3: Predictors in the lifestyle-based diabetes score by Simmons et al. (Simmons et al., 2007), the corresponding variables in the validation dataset, and description/question contained in the 45 and Up questionnaire (Sax Institute, 2019).

| **Predictors** | **Variables in validation dataset** | **Description/Question** |
| --- | --- | --- |
| Age | Age | Age based on date on baseline questionnaire and date of birth |
| Sex | Sex | Participant gender |
| Family history of diabetes: parents/siblings | Family history of diabetes: no cross 🡪 none; cross for mother, father, or siblings 🡪 parent or sibling; cross for mother OR father AND sibling 🡪 parent and sibling | Have your mother, father, brother(s) or sister(s) ever had (blood relatives only): diabetes 🡪 cross in box for mother or father or brother/sister indicates ‘yes’ |
| BMI (kg/m^2^) | Weight (in kg) and height (in cm) 🡪 BMI = weight [kg] / (height [cm]/100)^2^) | About how much do you weigh? How tall are you without shoes? |
| Antihypertensive drugs | Antihypertensive drugs | In the last month have you been treated for: high blood pressure? |
| Recreational physical activity (hours per week) | Minutes of moderate and vigorous physical activity (older version of questionnaire contained only minutes, new version minutes and hours) transformed into hours; all added together to get hours of exercise per week. | What do you estimate the total time that you spent doing this moderate physical activity in the last week was? (hours) |
|  |  | What do you estimate the total time that you spent doing this moderate physical activity in the last week was? (minutes) |
|  |  | What do you estimate the total time that you spent doing this vigorous physical activity in the last week was? (hours) |
|  |  | What do you estimate the total time that you spent doing this vigorous physical activity in the last week was? (minutes) |
| Green leafy vegetables (portions per day) | No information on green leafy vegetables, instead subsidised by raw vegetables. Assumption: one serve equals one portion. | About how many serves of raw vegetables do you usually eat each day? |
| Fruits (times per week) | Assumption: one serve equals one time. | About how many serves of fruit do you have each day? (excluding fruit juices) |
| Wholemeal/ brown bread (times per week) | Number of slices or pieces of brown or wholemeal bread. Assumption: one slice/piece equals one time. | About how many of the following do you usually eat: slices or pieces of brown/wholemeal bread each week (also include multigrain, rye bread, etc.) |

Abbreviation: BMI = body mass index.

**AUSDRISK score (Chen et al., 2010)**

Modifications required to fit the 45 and Up Study data:

- - Ethnicity: only Southern European, and Asian background included. With the ethics approval that we have obtained, we do not have access to information of Aboriginal and Torres Strait Islander or Pacific Islander status of participants. According to the 45 and Up Study baseline databook, 0.7% of baseline participants stated that they have Aboriginal status and 0.1% that they have Torres Strait Islander Status (1.8% missing values) (Sax Institute, 2011).
  - History of high blood glucose: no information available, everyone assumed to have no history of high blood glucose.
  - BMI used instead of waist circumferences.

Predictor assessment that differs to lifestyle-based model by Simmons et al.:

- - Ethnicity, question: “In which country were you born?” – if cross in box for any of the following:
    1. for Southern European background: Italy, Greece, Malta
    2. for Asian background: China, Lebanon, Philippines, Vietnam.
  - Parental history: as ‘family history’ above (Table S3), but without considering siblings.
  - Current smoker, question: “Are you a regular smoker now?” – if cross in box “Yes”.
  - Physical inactivity: as ‘recreational physical activity’ above (Table S3), but vigorous physical activity multiplied by 2.

## Statistical analysis methods and software

**Recalibration methods:**

1. Calibration-in-the-large:
   - Method according to Janssen et al. (Janssen et al., 2007; Janssen et al., 2009);
   - Intercept recalibrated by adding correction factor to adjust for differences in diabetes incidence between validation and derivation dataset;
   - Compute:

$correction factor=ln(\frac{incidence in validation set}{1-incidence in validation set}/\frac{mean predicted risk in validation set}{1-mean predicted risk in validation set})$.

1. Logistic calibration:
   - Method according to Harrell et al. (Harrell et al., 1996; Janssen et al., 2007);
   - Adjust calibration intercept and slope;
   - Compute:

$\ln\left( \frac{risk of T2DM}{1-risk of T2DM} \right)=a_{calibration}+\beta_{calibration}*linear predictor$

where $a_{calibration}$: calibration intercept, and $\beta_{calibration}$: calibration slope;

- Intercept and slope were calculated with val.prob-function from rms-package (Harrell, 2020).

**Test for significance of predictor variables:**

- For likelihood-ratio test: compute an analysis of deviance table for logistic regression model with deviance, degrees of freedom, and p-value using the anova-function.

**Model discrimination using pROC-package (Robin et al., 2011)**

- Calculate area under the receiver operating characteristic curve (AUC) with trapezoids (Fawcett, 2006)
- Calculate 95% confidence interval (CI) of the AUC according to DeLong’s method (DeLong et al., 1988)
- Bias-corrected AUC for refitted models (Harrell et al., 1996):
  - compute AUC of model on same validation dataset that was used to derive refitted model (apparent AUC: AUC_app_);
  - generate sample of size of the validation dataset with replacement from the latter;
  - fit model using bootstrap sample and compute AUC (AUC_boot_);
  - assess performance of the fit in original validation dataset (AUC_orig_);
  - calculate optimism (O_est_) in the fit from the bootstrap samples:
    $O_{est}= {AUC}_{boot}- {AUC}_{orig}$;
  - repeat steps 2 – 5 1000 times;
  - average optimism estimates to get overall optimism (O);
  - compute bias-corrected AUC: ${AUC}_{bias-corr}={AUC}_{app}-O$.

**Model calibration using rms-package (Harrell, 2020)**

- Grouped proportions vs. mean predicted probability in group;
- Linear logistic calibration fit;
- Smooth nonparametric fit: loess smoother using locally weighted polynomial regression (Austin and Steyerberg, 2014; Cleveland, 1979);
- Ideal line (i.e., intercept α = 0, slope β = 1) to indicate perfect calibration.

**Brier score for overall model performance using rms-package (Harrell, 2020)**

- Compute: $\sum{(y_{i}-p_{i})}^{2}/n$ , where y: observed outcome and p: predicted probability for subject i in dataset of size n (Steyerberg et al., 2010);
- Since incidence rate in validation dataset is 5%, value for non-informative model is:
  $0.05*\left( 1-0.05 \right)^{2}+\left( 1-0.05 \right)*{0.05}^{2}=0.0475$ 🡪 values range from 0 – 0.0475.

**List of all R-packages used in analysis:**

- haven,
- dplyr,
- mice,
- Hmisc,
- pROC,
- rms,
- ggplot2,
- naniar,
- MASS,
- sampling,
- grid,
- and further dependencies.

# Results

## Missing values

Table A.4: Comparison of the characteristics of individuals with any missing value and those with completely observed data.

| **Variables** | **Complete data (n = 74,220)** | **Missing data (n = 23,395)** | **p-value** |
| --- | --- | --- | --- |
| T2DM incidence in follow-up time ^a^ | 3,411 (4.6) | 1,330 (5.7) | <0.001 |
| Age, years ^b^ | 58.6 (52.3, 65.7) | 60.9 (53.8, 69.1) | <0.001 |
| Women ^a^ | 43,012 (58.0) | 12,272 (52.5) | <0.001 |
| Family history ^a^ | |  | 0.038 |
| Parent OR sibling with diabetes | 14,069 (19.0) | 4,261 (18.2) |  |
| Parent AND sibling with diabetes | 1,650 (2.2) | 535 (2.3) |  |
| BMI [kg/m^2^] ^a^ | | | <0.001 |
| < 25.00 | 29,993 (40.4) | 6,673 (38.6) |  |
| 25.00–27.49 | 17,395 (23.4) | 4,094 (23.7) |  |
| 27.50–29.99 | 12,295 (16.6) | 2,970 (17.2) |  |
| ≥ 30.00 | 14,537 (19.6) | 3,568 (20.6) |  |
| Antihypertensive drugs ^a^ | 15,054 (20.3) | 4,907 (21.0) | 0.023 |
| Physical activity ≥ 1 h/week ^a^ | 60,876 (82.0) | 14,491 (74.9) | <0.001 |
| Raw vegetables ≥ 1 serve/day ^a^ | 64,808 (87.3) | 11,142 (87.8) | 0.140 |
| Cooked vegetables ≥ 1 serve/day ^a^ | 72,580 (97.8) | 20,016 (98.3) | <0.001 |
| Fresh fruits ≥ 1 serve/day ^a^ | 69,459 (93.6) | 18,001 (92.6) | <0.001 |
| Brown bread ≥ 1 slice/day ^a^ | 65,539 (88.3) | 16,326 (85.0) | <0.001 |

^a^ n (%) and Pearson’s Chi-squared test with Yates’ continuity correction.
^b^ non-parametric: median (interquartile range) and Mann-Whitney U test.

Abbreviations: BMI = body mass index; T2DM = type 2 diabetes mellitus

**AUSDRISK score**

For external validation of the AUSDRISK score, we performed an independent imputation of missing values. The only additional variable with missing values was current smoking status with 23 missing observations. The missing values were imputed using logistic regression imputation.

Table A.5: Frequency of combinations of missing predictors.

| **Missing predictor** | **Number of participants** | **Percent (%) of participants** |
| --- | --- | --- |
| 0 | 74,220 | 76.0 |
| 1 | 18,180 | 18.6 |
| 2 | 3,298 | 3.4 |
| 3 | 598 | 0.6 |
| 4 | 1,159 | 1.2 |
| 5 | 149 | 0.2 |
| 6 | 11 | 0.0 |


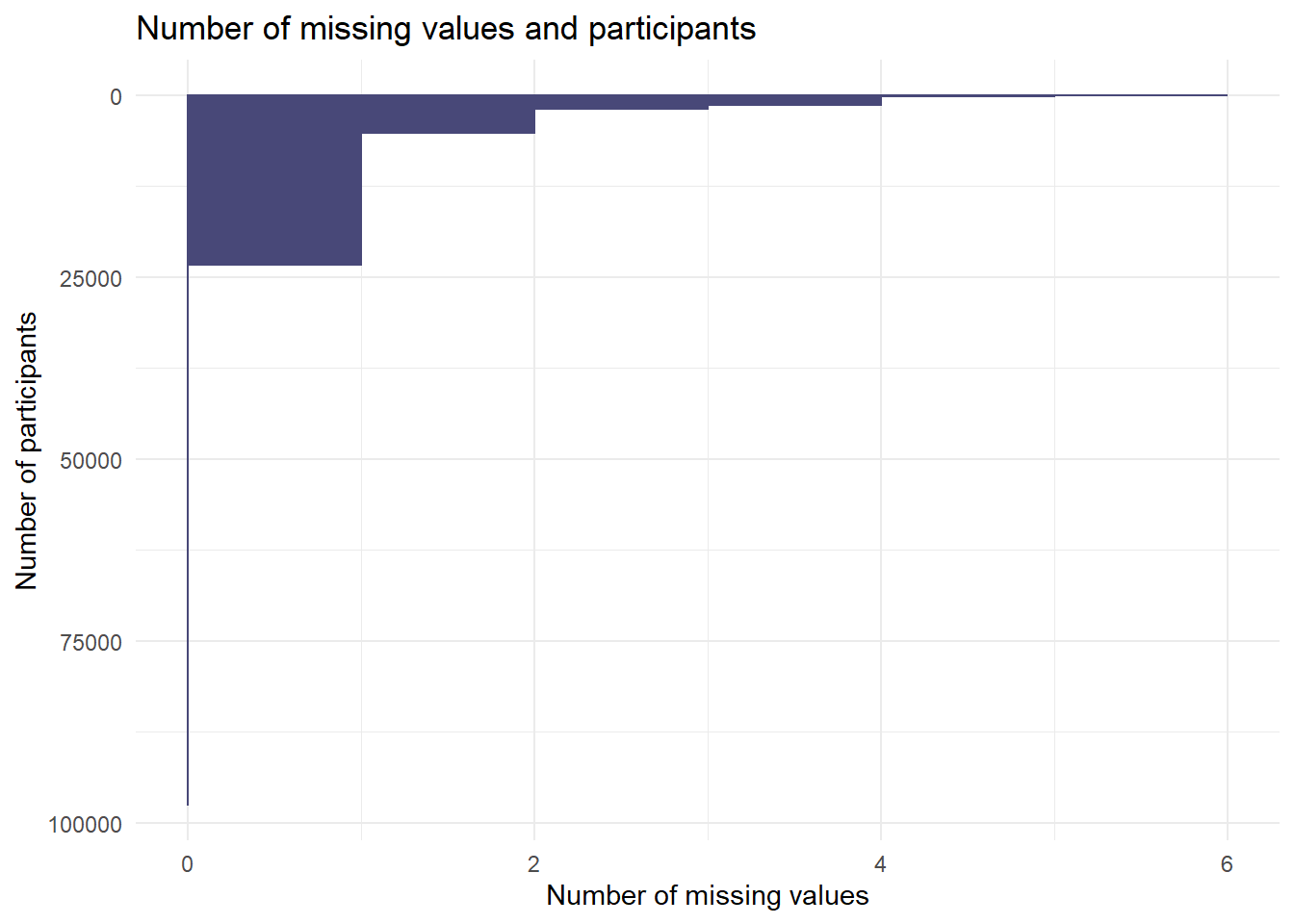


Figure A.1: Visualisation of the number of participants vs. the number of missing values.


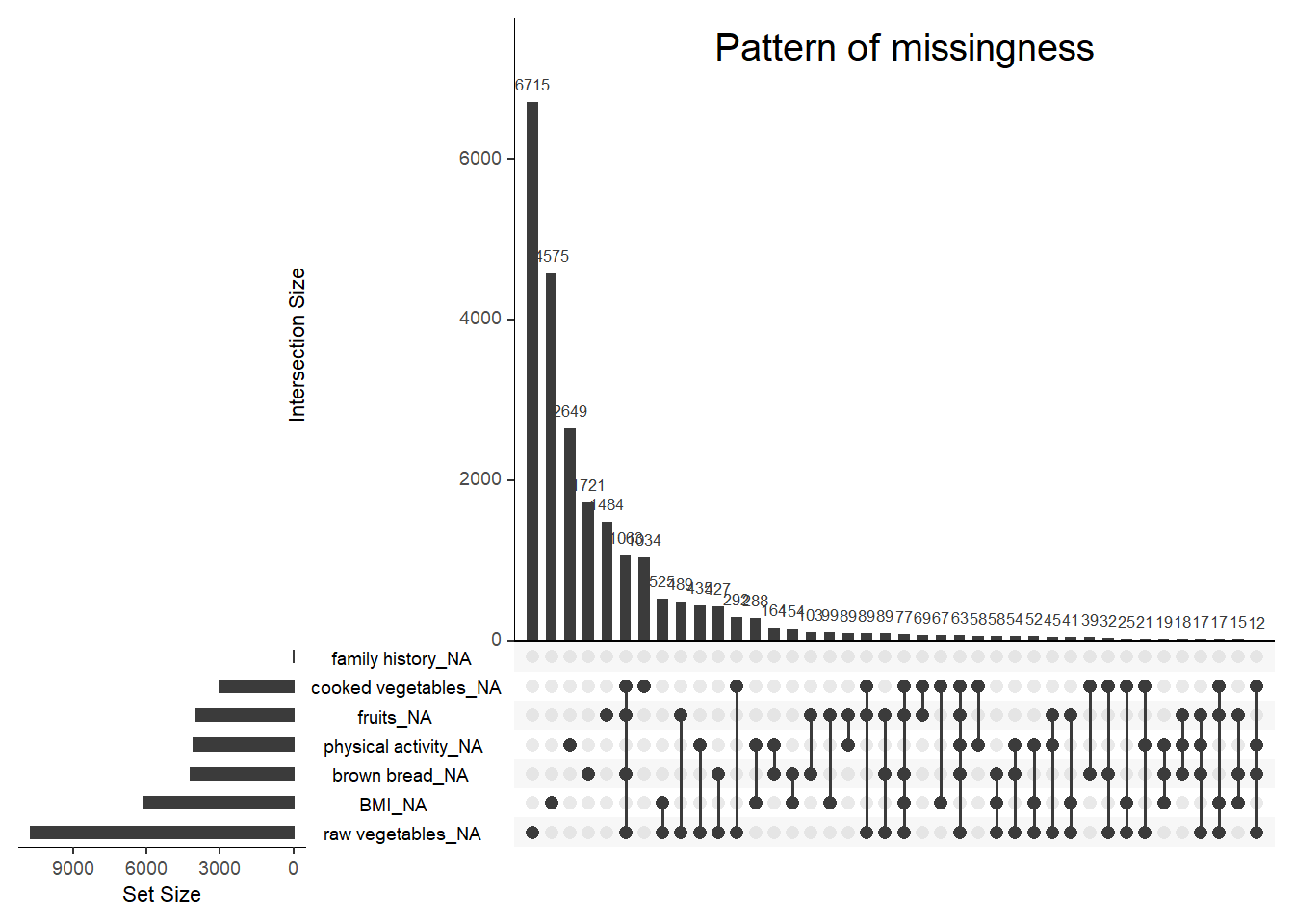


Figure A.2: Interactions between the variables with missing values.


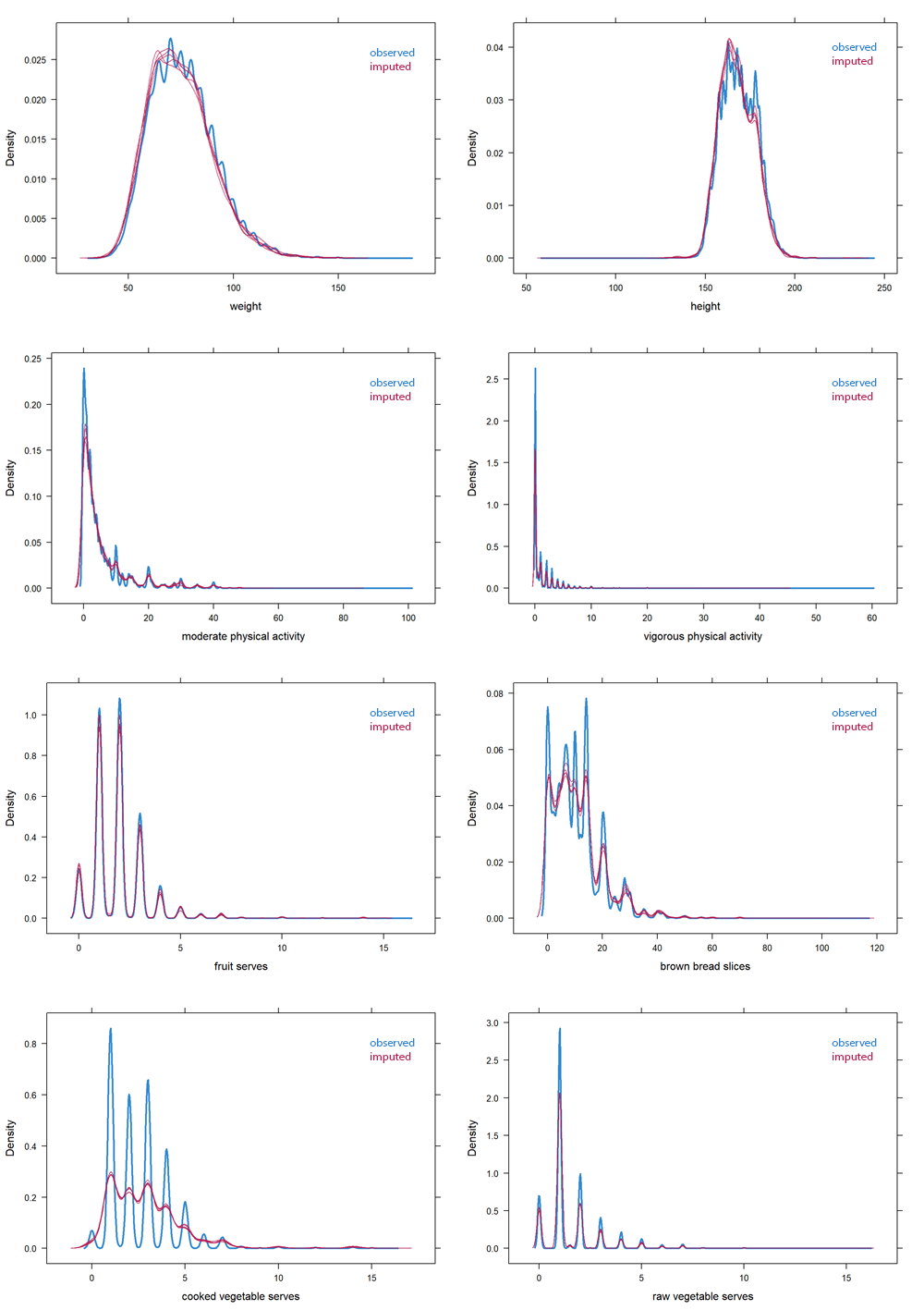


Figure A.3: Density plots of imputed (red, n = 5) and observed (blue) data.

## Performance of original model


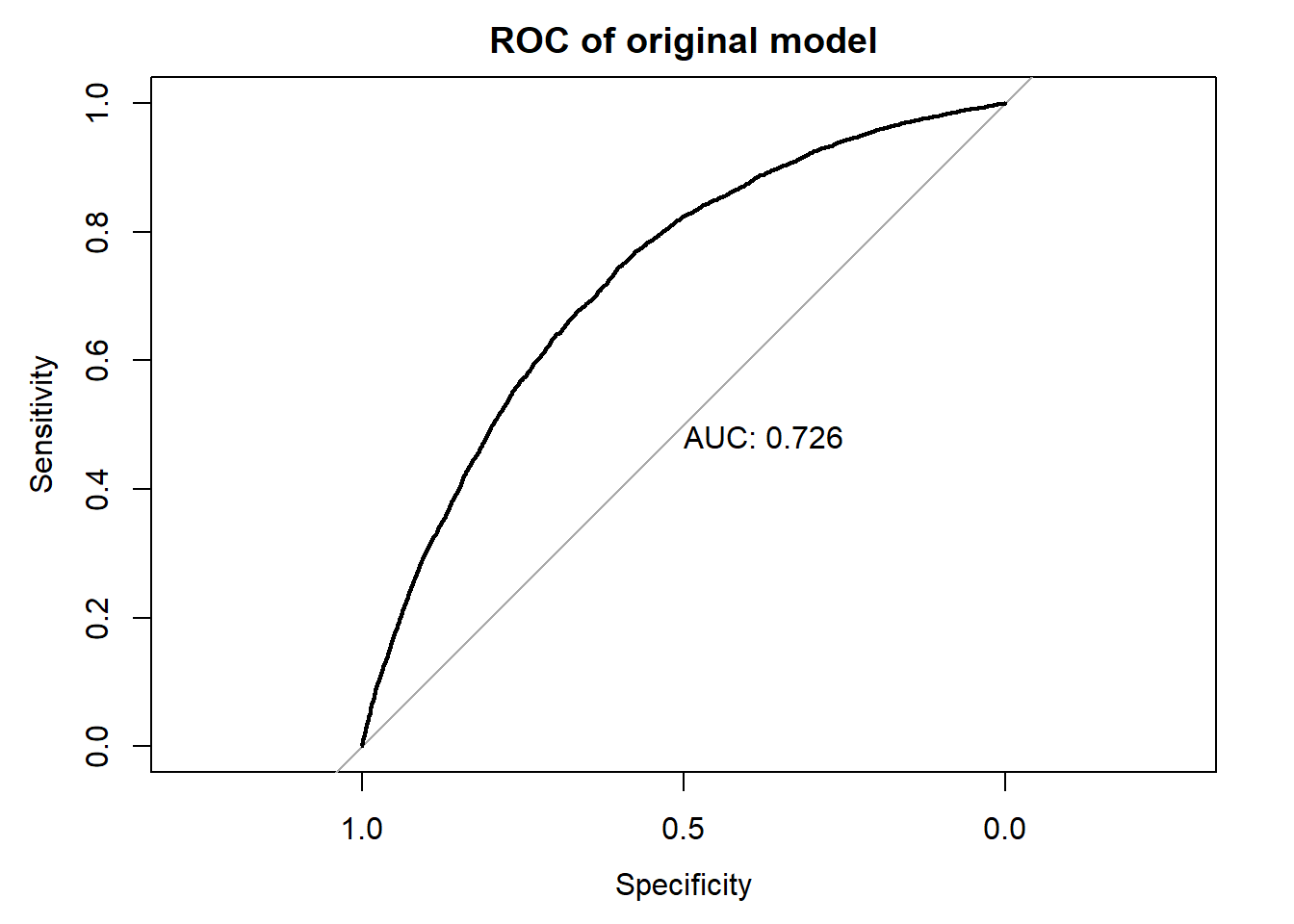


Figure A.4: Receiver operating characteristic curve of model 0 (original model).

## Specifications of updated models

Table A.6: The different models and their predictor variables with corresponding coefficients.

| Variables | Model 0 (original model) | Model 1 (cal.-in-large) | Model 2 (logistic cal.) | Model 3 (refitted) | Model 4 (refitted, changed predictor) | Model 5 (refitted, contin.) |
| --- | --- | --- | --- | --- | --- | --- |
| Intercept | -6.925 | -5.389 | -4.742 | -5.050 | -4.839 | -8.175 |
| Age [years] | 0.037 | 0.037 | 0.029 | 0.024 | 0.025 | 0.027 |
| Sex (reference: male) | -0.283 | -0.283 | -0.220 | -0.318 | -0.322 | -0.395 |
| Family history (reference: no family history)^a^ | 0.477  0.778 | 0.477  0.778 | 0.371  0.606 | 0.617  0.990 | 0.616  0.990 | 0.628  0.997 |
| BMI [kg/m^2^] (reference: BMI < 25.00)^b^ | 0.921  1.274  2.096 | 0.921  1.274  2.096 | 0.717  0.992  1.632 | 0.544  0.952  1.688 | 0.543  0.951  1.686 | 0.128 |
| Antihypertensive drugs (reference: no drugs) | 0.349 | 0.349 | 0.272 | 0.473 | 0.473 | 0.454 |
| Physical activity [hours/week]  (reference: < 1)^c^ | -0.388 | -0.388 | -0.302 | -0.237 | -0.238 | -0.010 |
| Vegetables [serves/day] (reference: < 1)^d^ | -0.539 | -0.539 | -0.420 | -0.091 | -0.288 | -0.008 |
| Fruits [serves/day] (reference: no fruits)^e^ | 0.055  -0.041 | 0.055  -0.041 | 0.043  -0.032 | -0.190  -0.110 | -0.196  -0.114 | -0.016 |
| Brown bread [slices/week] (reference: no brown bread)^f^ | -0.317  -0.085  -0.931  -0.459 | -0.317  -0.085  -0.931  -0.459 | -0.247  -0.066  -0.725  -0.357 | 0.062  -0.082  -0.004  -0.038 | 0.059 -0.086 -0.009 -0.042 | -0.001 |

Abbreviations: BMI = body mass index, cal. = calibration, contin. = continuous.

^a^ group 1: (Parent OR sibling with diabetes), group 2: (Parent AND sibling with diabetes)

^b^ group 1: BMI 25.00–27.49, group 2: BMI 27.50–29.99, group 3: BMI ≥ 30.00; for model 5: BMI 🡪 continuous

^c^ for model 5, physical activity [hours/week] = moderate physical activity [hours/week] + 2 * vigorous physical activity [hours/week] 🡪 continuous

^d^ for model 0, 1, and 2: serves of raw vegetables; for model 3 and 4: serves of raw + cooked vegetables; for model 5: serves of raw + cooked vegetables 🡪 continuous

^e^ group 1: more than 0 but less 1 serve of fruits per day, group 2: at least 1 serve of fruit per day; for model 5: serves of fruit 🡪 continuous

^f^ group 1: more than 0 and up to 1 slice per week, group 2: more than 1 but less than 5, group 3: 5 or more but less than 7 slices, group 4: 7 slices or more per week; for model 5: slices of brown bread 🡪 continuous

## Performance of the refitted models


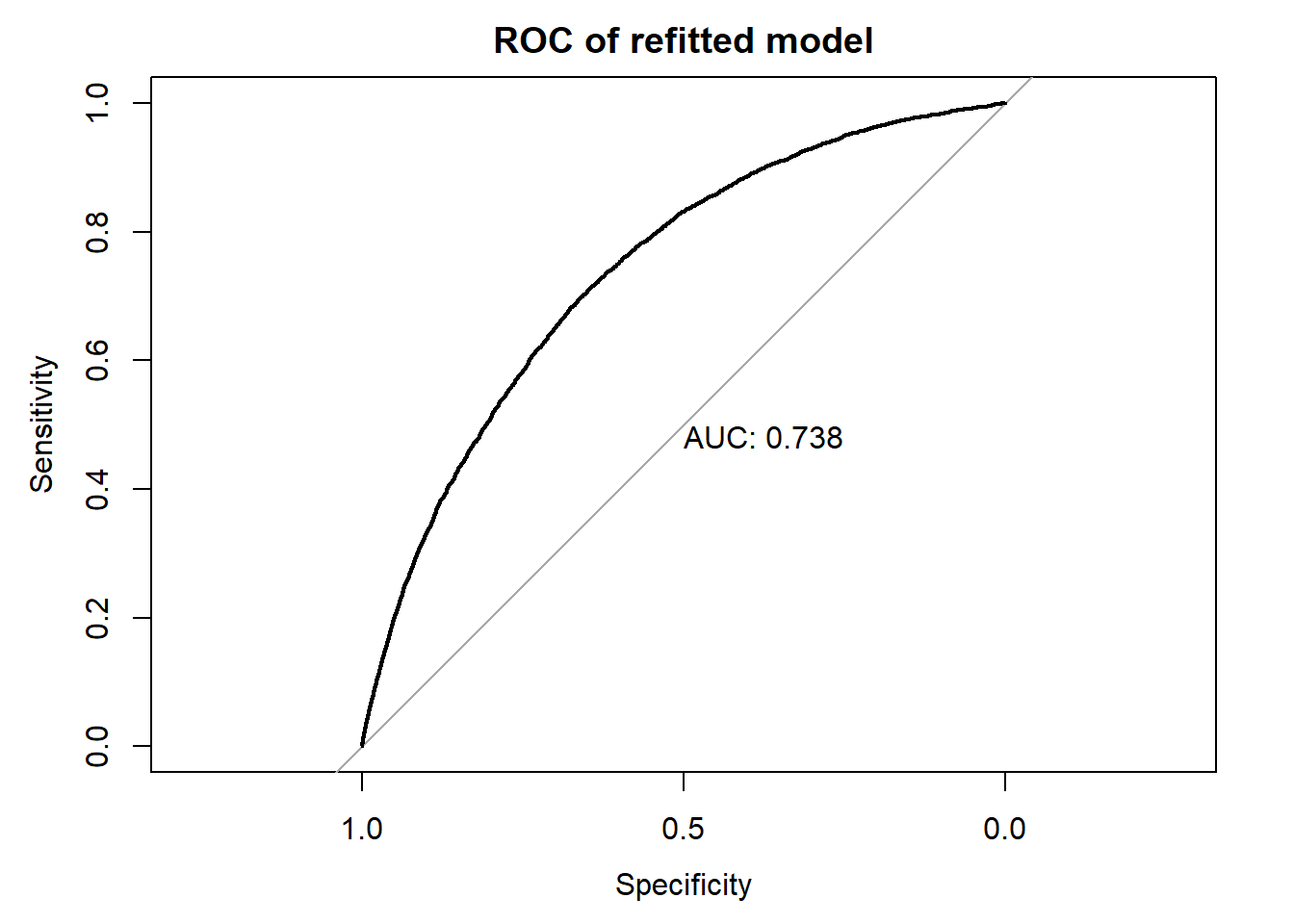


Figure A.5: Receiver operating characteristic curve of models 3 (refitted) and 4 (refitted and changed predictor).


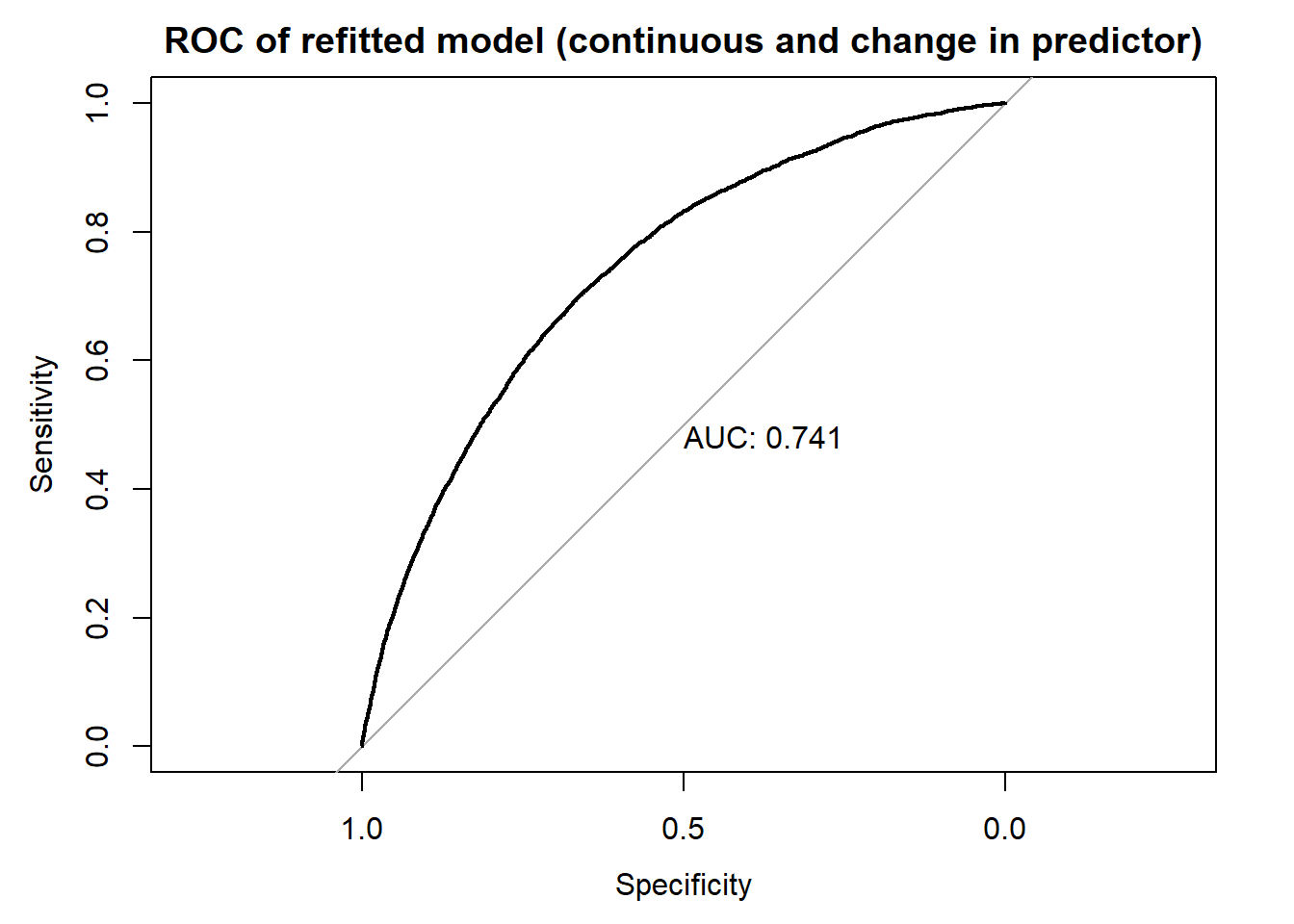


Figure A.6: Receiver operating characteristic curve of model 5 (refitting with numerical predictors as continuous).


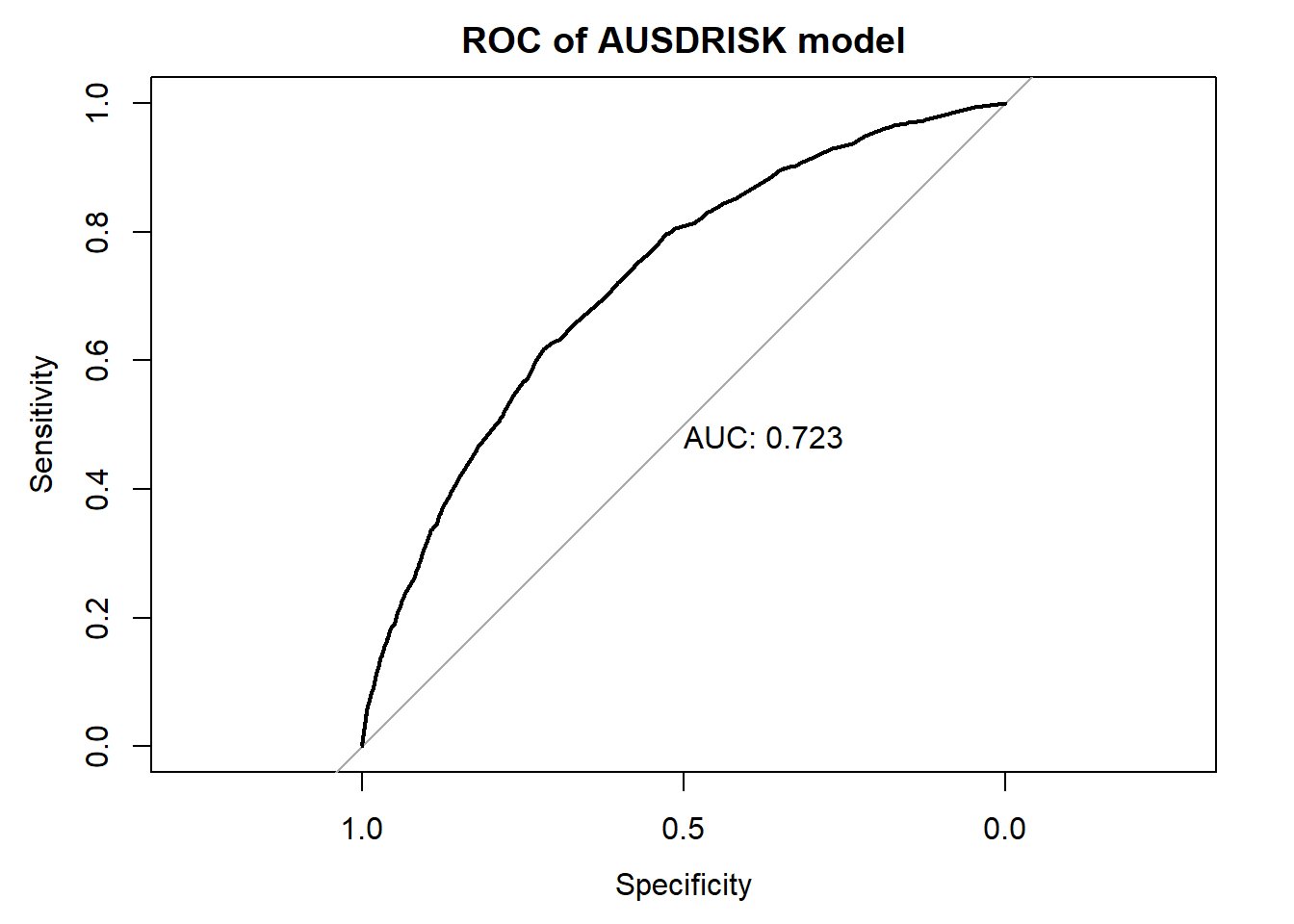


Figure S7: Receiver operating characteristic curve of the AUSDRISK model.

# References

Austin, P.C., Steyerberg, E.W., 2014. Graphical assessment of internal and external calibration of logistic regression models by using loess smoothers. Stat. Med. 33:517-35.

Australian Government Department of Health, 2020a. The Pharmaceutical Benefits Scheme - Browse by body system. Commonwealth of Australia, Canberra, Australia.

Australian Government Department of Health, 2020b. Schedule of pharmaceutical benefits: General pharmaceutical schedule - effective 1 September 2020, Canberra, Australia, p. 1079.

Australian Institute of Health and Welfare, 2009. Diabetes prevalence in Australia:  An assessment of national data sources Diabetes Series. Australian Institute of Health and Welfare, Canberra, Australia, p. 41.

Australian Institute of Health and Welfare, 2020. Diabetes web pages data tables, in: Australian Institute of Health and Welfare (Ed.), Canberra, Australia.

Chen, L., Magliano, D.J., Balkau, B., Colagiuri, S., Zimmet, P.Z., Tonkin, A.M., Mitchell, P., Phillips, P.J., Shaw, J.E., 2010. AUSDRISK: An Australian type 2 diabetes risk assessment tool based on demographic, lifestyle and simple anthropometric measures. Med. J. Aust. 192:197-202.

Cleveland, W.S., 1979. Robust locally weighted regression and smoothing scatterplots. J. Am. Stat. Assoc. 74:829-36.

Comino, E.J., Tran, D.T., Haas, M., Flack, J., Jalaludin, B., Jorm, L., Harris, M.F., 2013. Validating self-report of diabetes use by participants in the 45 and up study: a record linkage study. BMC Health Serv. Res. 13.

Commonwealth of Australia, 2003. Schedule of pharmaceutical benefits for approved pharmacists and medical practitioners. Australian Government, Canberra, Australia, p. 439.

DeLong, E.R., DeLong, D.M., Clarke-Pearson, D.L., 1988. Comparing the areas under two or more correlated receiver operating characteristic curves: A nonparametric approach. Biometrics 44:837-45.

Fawcett, T., 2006. An introduction to ROC analysis. Pattern Recognit. Lett. 27:861-74.

Harrell, F.E.J., Lee, K.L., Mark, D.B., 1996. Multivariable prognostic models: Issues in developing models, evaluating assumptions and adequacy, and measuring and reducing errors. Stat. Med. 15:361-87.

Harrell, F.J., 2020. rms, v6.1-0 ed. Harell, Frank Jr., CRAN repository.

Janssen, K.J.M., Moons, K.G.M., Kalkman, C.J., Grobbee, D.E., Y., V., 2007. Updating methods improved the performance of a clinical prediction model in new patients. J. Clin. Epidemiol. 61:76-86.

Janssen, K.J.M., Vergouwe, Y., Kalkman, C.J., Grobbee, D.E., Moons, K.G.M., 2009. A simple method to adjust clinical prediction models to local circumstances. Can. J. Anaesth. 56:194.

Robin, X., Turck, N., Hainard, A., Tiberti, N., Lisacek, F., Sanchez, J.-C., Müller, M., 2011. pROC: An open-source package for R and S+ to analyze and compare ROC curves. BMC Bioinformatics 12:77.

Sax Institute, 2011. The 45 and Up Study baseline questionnaire data book. Sax Institute, Sydney, Australia, p. 26.

Sax Institute, 2019. Researcher toolkit - Sax Institute. Sax Institute, Sydney, Australia.

Simmons, R.K., Harding, A.H., Wareham, N.J., Griffin, S.J., 2007. Do simple questions about diet and physical activity help to identify those at risk of type 2 diabetes? Diabet. Med. 24:830-35.

Steyerberg, E.W., Vickers, A.J., Cook, N.R., Gerds, T., Gonen, M., Obuchowski, N., Pencina, M.J., Kattan, M.W., 2010. Assessing the performance of prediction models: A framework for traditional and novel measures. Epidemiology 21:128-38.
